# SnO_2_-Based Memory Device with Filamentary Switching Mechanism for Advanced Data Storage and Computing

**DOI:** 10.3390/nano13182603

**Published:** 2023-09-21

**Authors:** Muhammad Ismail, Chandreswar Mahata, Myounggon Kang, Sungjun Kim

**Affiliations:** 1 Division of Electronics and Electrical Engineering, Dongguk University, Seoul 04620, Republic of Koreachandreswar@gmail.com (C.M.); 2 Department of Electronics Engineering, Korea National University of Transportation, Chungju-si 27469, Republic of Korea

**Keywords:** tin oxide, resistive switching, multilevel resistance states, Schottky emission, neuromorphic system

## Abstract

In this study, we fabricate a Pt/TiN/SnO_x_/Pt memory device using reactive sputtering to explore its potential for neuromorphic computing. The TiON interface layer, formed when TiN comes into contact with SnO_2_, acts as an oxygen vacancy reservoir, aiding the creation of conductive filaments in the switching layer. Our SnO_x_-based device exhibits remarkable endurance, with over 200 DC cycles, ON/FFO ratio (>20), and 10^4^ s retention. Set and reset voltage variabilities are impressively low, at 9.89% and 3.2%, respectively. Controlled negative reset voltage and compliance current yield reliable multilevel resistance states, mimicking synaptic behaviors. The memory device faithfully emulates key neuromorphic characteristics, encompassing both long-term potentiation (LTP) and long-term depression (LTD). The filamentary switching mechanism in the SnO_x_-based memory device is explained by an oxygen vacancy concentration gradient, where current transport shifts from Ohmic to Schottky emission dominance across different resistance states. These findings exemplify the potential of SnO_x_-based devices for high-density data storage memory and revolutionary neuromorphic computing applications.

## 1. Introduction

Memristors have garnered substantial attention due to their prospective contributions to high-density nonvolatile memory and neuromorphic computing [1,2]. These devices offer operational simplicity and low energy consumption, making them conducive to swift read–write processes [3,4,5]. Additionally, memristors exhibit intriguing potential in terms of scalability, density enhancement, cost effectiveness, and compatibility with complementary metal oxide semiconductor (CMOS) technology [4,6]. Neuromorphic electronic synapses, fundamental elements in constructing efficient neuromorphic computing systems, have harnessed the capability to replicate critical bio-synaptic functions through conductance modulation [7,8]. Neuromorphic computing, a burgeoning paradigm, has emerged as a solution to the bottlenecks posed by conventional von Neumann computers, aligning well with the requirements of future computing systems [9]. It is widely acknowledged that the fundamental operation of memristors hinges on the migration of oxygen ions. In this dynamic landscape, valence change memory (VCM) and electrochemical metallization memory (ECM) stand out as pivotal mechanisms [10]. ECM memristors orchestrate the transfer of metal cations through oxidation of the active metal in the top electrode (TE) under bias voltage, resulting in the creation of a metallic filament via cation reduction at the inert electrode [11].

However, ECM memristors grapple with challenges encompassing inconsistent resistive switching (RS), endurance limitations, and long-term retention concerns arising from the stochastic nucleation and unregulated growth of conductive filaments, thereby posing significant impediments [12]. In contrast, VCM memristors orchestrate the creation or rupture of an oxygen-deficient filament through voltage application, driving the migration of oxygen anions/vacancies and modifying the Schottky barrier. A comprehensive understanding of these mechanisms emerges as crucial for the effective development of reliable and efficient memristive devices catering to an array of applications. VCM has garnered substantial attention due to its reliance on reactive metal electrodes like Al, Zr, Ti, TaN, TiN, etc. [13,14,15,16,17,18]. This selection stems from their innate tendency to readily oxidize upon interaction with oxide materials, fostering the creation of an interface layer. This layer performs a dual role by serving as a repository for oxygen vacancies while facilitating the intricate process of conductive filament formation within the switching layer. These insights corroborate previous findings that underscore the pivotal role of the interface layer in governing memristor performance, further cementing the relevance of these electrode materials in advancing memristor technology.

Previous studies reflect a discernible trend in utilizing reactive metal electrodes to engender interface layers in memristor devices. For instance, the work conducted by Li et al. [19] delved into the integration of a titanium nitride (TiN) electrode with TaO_x_, revealing the emergence of an interface layer rich in oxygen vacancies. This layer’s role as an oxygen vacancy repository played a pivotal role in enhancing the memristor switching characteristics. Similarly, in our previous investigation [20], we explored tantalum (Ta) as a reactive metal electrode, revealing the establishment of an interface layer through the process of tantalum oxidation upon contact with ZnSnO. This layer not only bolstered stability, but also facilitated controlled conductive filament generation. These instances reiterate the recurrent theme in literature, where the astute selection of reactive metal electrodes—such as Ti, TiN, and Ta, among others—consistently led to interface layer formation. These layers, abundant in oxygen vacancies, assumed a pivotal role in optimizing conductive filament establishment within the switching layer. These insights corroborate the pivotal role of interface engineering through reactive metal electrodes in optimizing memristor performance, aligning seamlessly with the current study’s observations. Transition metal oxides (TMOs) encompass a captivating cohort of materials, profoundly explored for their roles in resistive RS memory. This category includes a diverse range of oxide materials, including TiO_2_, ZrO_2_ [21], Ta_2_O_5_ [22], HfO_2_ [23], CeO_2_ [24], and Al_2_O_3_ [25], alongside semiconducting counterparts like ZnO [26], ITO [27], and, notably, SnO_2_ [28]. These materials have been vigorously examined for their suitability as RS layers within resistive random-access memories (RRAMs). In addition, Ginnaram et al. [29] demonstrate Hf/Si interface key role in enhancing RRAM durability and performance, achieving > 4000 DC cycles and >2 × 10^9^ P/E endurance at low current and fast switching speeds.

Recently, semiconductors materials like IGZO (Indium Gallium Zinc Oxide) [30], SnO_x_ (Tin Oxide) [31] and ZnO (Zinc Oxide) [32] have emerged as promising candidates for achieving gradual modulation of conductance. This unique properties of these materials make them highly advantageous for the development of neuromorphic systems, particularly when employed as synaptic devices. The gradual conductance modulation characteristic exhibited by these semiconductors holds significant potential for advances in neuromorphic computing. This capability allows for the emulation of synaptic behavior in a more precise and efficient manner, mirroring the plasticity and adaptability of biological synapses. As a result, these materials play a crucial role in the realization of advanced neuromorphic systems, opening up exciting possibilities for artificial intelligence and cognitive computing applications. Among these contenders, SnO_x_ has emerged as a particularly intriguing semiconducting material, attracting substantial interest within the realm of oxide electronics. While extensive research has been dedicated to scrutinizing TMO-based RS memory, the exploration of SnO_2_-based RS memory as a focal point has remained limited to a handful of studies, often concentrating on SnOx-based RS memory.

In this study, a TiN/SnO_x_/Pt memory device was fabricated using reactive sputtering to investigate both resistive switching and biological characteristics for a neuromorphic system. The memory devices based on SnO_x_ exhibited analog switching behavior, demonstrating exceptional durability for up to 200 DC cycles, favorable multi-state retention properties, and optimized variability with respect to set and reset voltages (1.98% and 0.9%, respectively). The ability to achieve multilevel switching characteristics was demonstrated by manipulating the reset voltage and current limitation, a crucial aspect for neuromorphic computing. Furthermore, the fundamental synaptic functions such as potentiation and depression were successfully emulated. The memory device exhibited Schottky and ohmic conduction mechanisms in the high-resistance state (HRS) and low-resistance state (LRS). To elucidate the switching mechanism of the SnO_x_-based memory devices, a filamentary model based on oxygen vacancies was proposed.

## 2. Experimental Procedures

**Figure 1:**
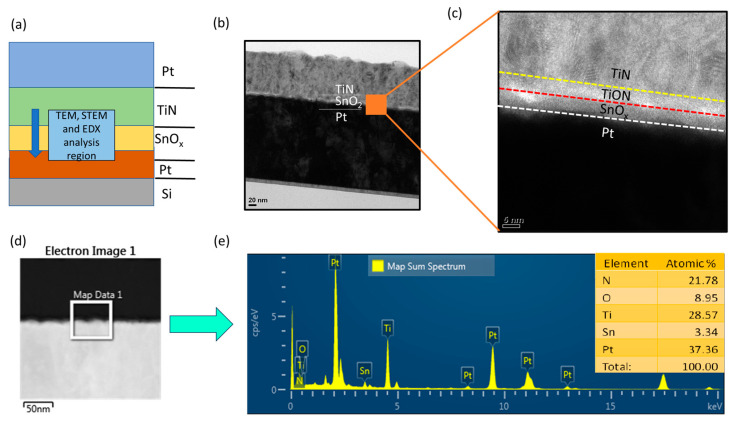
(**a**) Architecture of the Pt/TiN/SnO_x_/Pt memory device. The insets showcase the transmission electron microscopy (TEM) analyses that were performed after the focused ion beam (FIB) analysis. (**b**) Representative cross-sectional TEM image of a TiN/SnO_x_/Pt memory device. (**c**) Magnified TEM view highlighting the formation of the TiON interface layer in contact between the TiN top electrode and the SnO_x_ switching layer. (**d**,**e**) Cross-sectional STEM image and EDS spectra with inset of the stoichiometry ratio of elements present in the Pt/TiN/SnOx/Pt memory device.

The fabrication process for the Pt/TiN/SnO_x_/Pt memory device involved sequential steps. Initially, a 5 nm thick SnOx switching layer was deposited onto a pre-prepared silicon (Si) substrate using Pt deposition followed by radio frequency (RF) reactive sputtering. The sputtering process involved the use of a Sn source target and a gas mixture of Argon (Ar) at a flow rate of 19 standard cubic centimeters per minute (sccm) and Oxygen (O_2_) at a flow rate of 1 sccm, conducted at room temperature under a pressure of 5 millitorr (mTorr) with an RF sputter power of 50 watts (W). Following this, a 50 nm thick TiN top electrode (TE) was deposited using a Ti metal target in a gas mixture of 19 sccm Ar and 1 sccm N_2_, with a chamber pressure of 3 mTorr and a DC power of 280 W. Finally, square-shaped Pt/TiN (50/50 nm) top electrodes (TEs) with areas measuring 100 × 100 μm^2^ were deposited through a lift-off process using DC sputtering. The TiN TE was deposited using a Ti metal target in a gas mixture of 19 sccm Ar and 1 sccm N_2_, with a chamber pressure of 3 mTorr and a DC power of 280 W. To protect the TiN layer from oxidation, Pt capping layer was deposited by sputtering in an atmosphere of 20 sccm argon (Ar), under a chamber pressure of 6 mTorr and a DC power of 280 W. The electrical characteristics of the Pt/TiN/SnOx/Pt structure were assessed using a Keithley 4200-SCS semiconductor parameter analyzer in DC mode, with the 4225-PMU ultra-fast pulse module, applying bias to the top electrode (TiN) while keeping the bottom electrode (Pt) grounded. Microstructure analysis was performed using High-Resolution Transmission Electron Microscopy (HRTEM, KANC, Suwon, Republic of Korea) with Scanning Transmission Microscopy (STEM) and Energy-Dispersive X-ray Spectroscopy (EDS) for detailed examination of the device’s structural properties, as shown in Figure 1a.

## 3. Results and Discussion

Figure 1a illustrates the schematic representation of the Pt/TiN/SnO_x_/Pt memory device structure. The insets included in the figure provide insights into the transmission electron microscopy (TEM) analyses, which were carried out following the focused ion beam (FIB) analysis. Transitioning to Figure 1b, a high-resolution TEM image showcases a cross-sectional view of the TiN/SnO_x_/Pt memory device architecture. Further elaboration is offered in Figure 1c, where a magnified TEM view focuses on the marked region from Figure 1b. Figure 1d,e presents scanning transmission electron microscopy (STEM) images in conjunction with energy-dispersive X-ray spectroscopy (EDX) results for the Pt/TiN/SnOx/Pt memory device. Within Figure 1e, the insets illustrate the stoichiometric ratios of individual elements present in the Pt/TiN/SnOx/Pt memory device. The EDX image is instrumental in confirming the presence of all elemental peaks within the spectra, providing crucial information about the chemical composition of the device. Importantly, the SnO_x_ film displayed within the structure demonstrates an amorphous nature. The approximate thickness of the SnO_x_ and TiON layers is measured at 5 nm and 2 nm, respectively. This particular thickness distribution can be attributed to the heightened reactivity of titanium (Ti) with oxygen (O_2_). The reaction between Ti and O_2_ (Gibb’s free energy −883.32 kJ/mol at 300 K) [33] gives rise to the formation of a TiON layer [34] during the deposition of SnO_2_, a phenomenon facilitated by the inherent tendency of Ti to react with oxygen. This intricate process potentially contributes to the formation of a somewhat defective SnO_x_ switching layer. Comparatively, the TiON layer exhibits enhanced electrical conductivity when contrasted with the SnO_x_ layer. This conductivity disparity within the TiON interface layer provides a conducive environment for the initiation and rupture of conductive filaments. These filaments play a pivotal role in the switching behavior of the device. Furthermore, the presence of a series of TiON layers in conjunction with the SnO_x_ material contributes significantly to the enhancement of switching memory characteristics.

**Figure 2:**
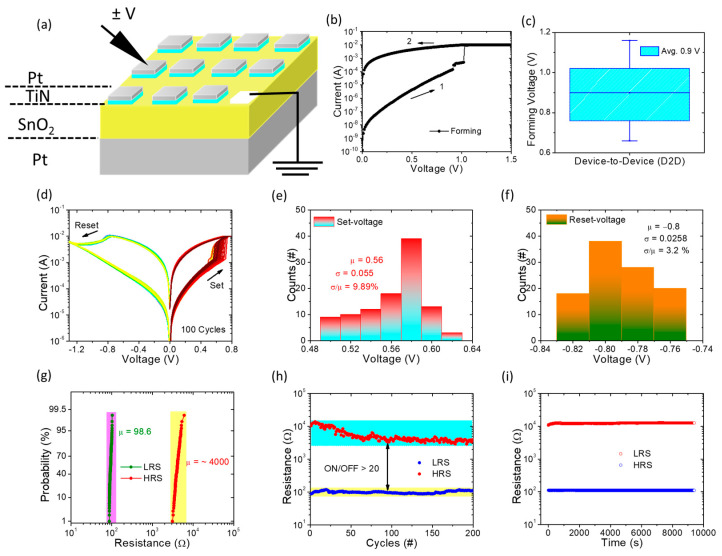
(**a**) Schematic layout of fabricated Pt/TiN/SnO_x_/Pt memory device with measurement configuration. (**b**) Typical current–voltage (I-V) characteristics of forming process. (**c**) Statistical distribution of forming voltage for 20 devices. (**d**) I-V curves of bipolar switching for 100 cycles. (**e**,**f**) Statistical distribution of set and reset voltages. (**g**) Cumulative probability distribution of R_LRS_ and R_HRS_, respectively. (**h**) Distribution of LRS and HRS over 200 DC operation cycles. (**i**) Retention times recorded at LRS and HRS for 10,000 s using a read voltage of 0.1 V.

Figure 2a illustrates the schematic layout of the TiN/SnO_x_/Pt memory device along with the measurement system. In this diagram, we can observe that all external biases are applied to the top electrode, while the bottom electrode remains grounded. This arrangement is crucial for the functioning of the memory device. It has been established that an initial forming process is necessary to initiate the resistive switching (RS) characteristics, which are essential for the device’s operation. As depicted in Figure 2b, the forming process of our memory device can be observed, wherein a current compliance (CC) of 10 mA is applied. As voltage is increased, the current through the memory device also increases, and it undergoes an abrupt switch at 1.05 V. This switch leads the memory device to transition from an initial state of high resistance to a state of low resistance. To ensure the device safety and prevent hard breakdown, the CC is limited. Additionally, Figure 2c presents the statistical results from 20 randomly selected memory devices used for measurement. These results reveal that the average forming voltage is approximately 0.9 V.

Subsequently, a sequence of 100 consecutive cycles of direct-current (DC) sweeping is conducted, as depicted in Figure 2d. The results vividly illustrate the consistent and replicable DC switching cycles exhibited by the memory devices during both positive set and negative reset operations. The statistical representation of the set and reset voltages from the data set of 100 current–voltage (IV) cycles is depicted in Figure 2e,f. Notably, the average set voltage is measured at 0.56 V, accompanied by a coefficient of variation of 9.89%. Meanwhile, the average reset voltage registers at −0.79 V, with a coefficient of variation of 3.2%. This distinct behavior can be attributed to the formation of a thin TiON interface layer, which effectively confines the conductive filament [35]. This confinement results in minimal variance, thereby ensuring heightened stability and remarkable consistency, largely due to the optimized generation of oxygen vacancies during the course of switching cycles. Furthermore, Figure 2g plots the cumulative distribution of resistance (Low Resistance State (LRS) and High Resistance State (HRS)) at a read voltage of 0.1 V across 100 cycles. The calculated average values for LRS and HRS are approximately 98.6 and 4000, respectively.

Additionally, the DC endurance performance is subjected to testing through C2C operations, as demonstrated in Figure 2h. Impressively, the memory device exhibits resilience over 500 DC cycles without any overlap between LRS and HRS states. Notably, the ON/OFF ratio surpasses 20, a notable achievement considering that the standard value is 10. This elevation in the ON/OFF ratio indicates that our devices fulfill the foundational requisites for nonvolatile memory applications. Moreover, Figure 2i offers insight into the retention properties of the memory devices by tracing current as a function of time for up to 10^4^ s at room temperature. Notably, no discernible degradation or fluctuation in resistance is observed in the LRS and HRS states. This robustness implies that the resistances of the memory devices remain unaffected even during continuous pulse read operations. In total, the TiN/SnO_x_/Pt memory device exhibits quintessential bipolar switching behaviors characterized by remarkable stability and reliability. This performance augments its potential for application in the realms of nonvolatile memory and neuromorphic computing.

**Figure 3:**
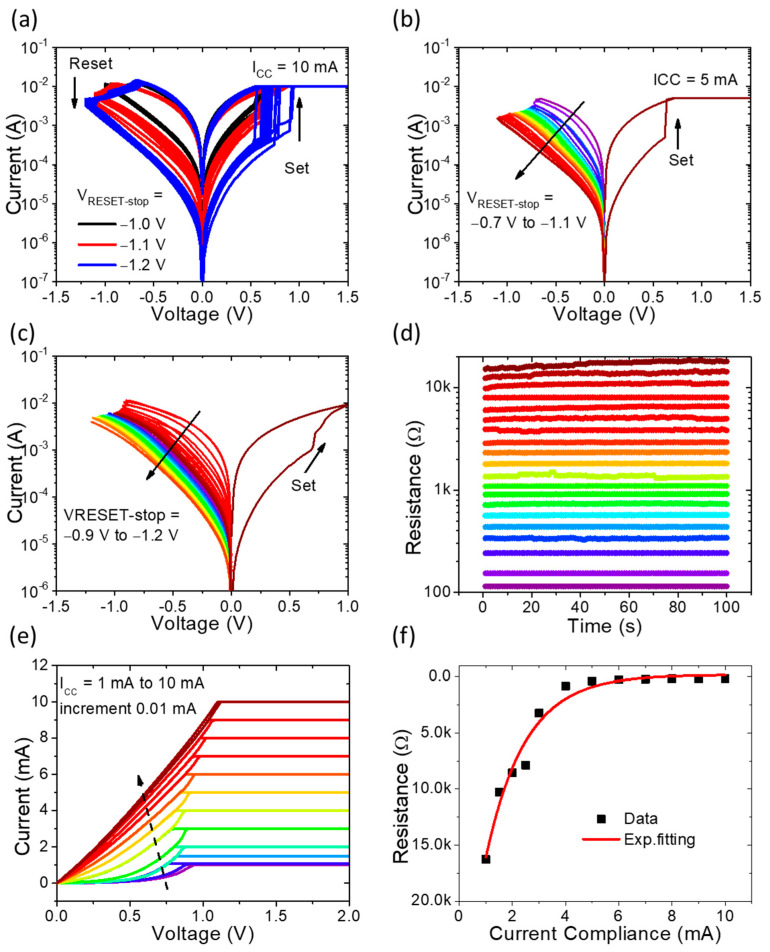
The multilevel switching capability of the Pt/TiN/SnO_x_/Pt memory device. (**a**) The 50 I-V curves of 50 continuous DC cycles with reset voltages at −1.0 V, −1.1 V, −1.2 V. (**b**) The abrupt set and multistep reset processes when increasing the reset voltage from −0.7 V to −1.1 V under 5 mA. (**c**) The gradual set and multistep reset processes when increasing the reset voltage from −0.9 V to −1.2 V under 10 mA. (**d**) Multilevel retention characteristics with a read at 0.1 V during 100 s for 20 different reset voltages. (**e**) Consecutive DC sweeping with different CC from 1 mA to 10 mA during the set process. (**f**) The 12 different resistance states extracted from Figure 3f.

The literature reports [36,37,38] the substantial potential of controllable switching characteristics in enhancing the construction of neural morphological computing systems. In the realm of biology, distinct resemblances exist between the attributes of synapses and memristor devices based on RRAM. The mouldability of the configuration through gradual reset voltage modulation and compliance current limitation has emerged as a pivotal determinant for the deployment of memristors in neuromorphic computing. Reset voltage modulation and compliance current limitation can be employed consecutively on the electrode to achieve resistance modulation, akin to biological synapse weight enhancement and inhibition serving as a means to stimulate synaptic plasticity. The reset stop voltage is progressively increased from −1.0 V to −1.2 V, leading to the acquisition of three distinct stable resistance states, as depicted in Figure 3a. Evidently, the ON/OFF ratios of memory devices are augmented with increasing reset stop voltage [39]. To further elucidate the analog switching characteristics, minor increments in the reset voltage are showcased under compliance currents of 5 mA and 10 mA, illustrated in Figure 3b,c, respectively. In these instances, the reset voltage is increased by a minimal step of 0.01 V. As an outcome of the meticulously controlled reset behavior exhibited by the memory devices, resistance values surge in response to increasing reset values, facilitating the attainment of multiple resistance levels [40]. Moreover, the maintenance of multilevel resistance states over a satisfactory retention time is achieved, as showcased in Figure 3d. These multilevel resistance states are induced through successive negative voltage sweeps spanning from −0.9 V to −1.2 V. Subsequent to each sweep cycle, the device’s resistance states are tracked by a sequence of 0.1 V reading pulses, spaced at intervals of 0.1 pulses. Furthermore, in the range of compliance currents varying from 1 mA to 10 mA, the corresponding current–voltage (I-V) curves, along with twelve distinct resistance states, are presented in Figure 3e,f, respectively.

**Figure 4:**
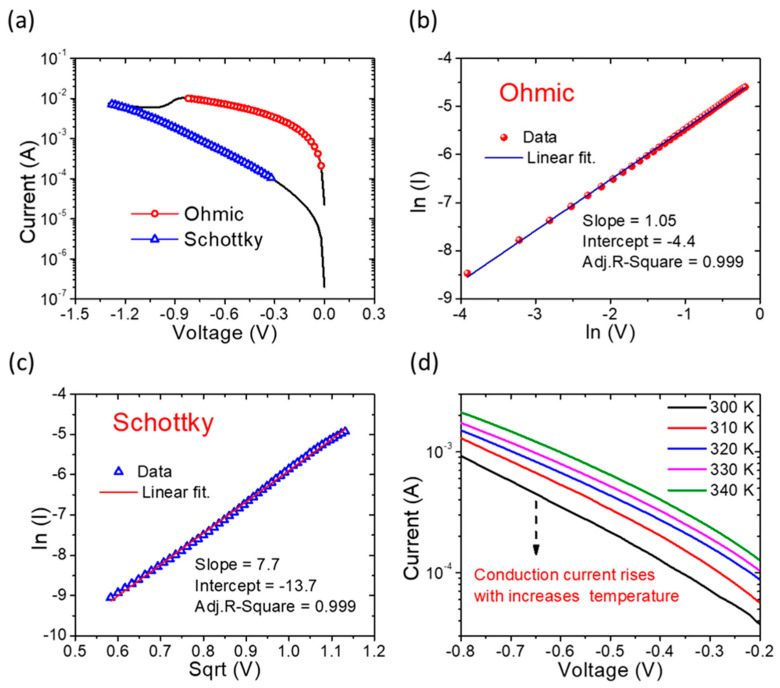
Fitting plot of Pt/TiN/SnO_x_/Pt memory device. (**a**) IV curve of the reset process; (**b**) LRS, ohmic conduction; (**c**) high-field region of the HRS, Schottky emission; (**d**) temperature dependence curves of the switching mode in the temperature range of 300 K to 340 K.

The analysis of the current conduction in the LRS and HRS of the I-V curve during the reset process involved fitting with respect to the ohmic conduction and Schottky emission mechanisms, respectively (Figure 4a). In the context of LRS fitting results, a linear correlation between Ln(I) and Ln(I) within the range of −0.02 V to −0.82 V was identified, as depicted in Figure 4b. The curved slope, measuring 1.05, was derived from Equations (1) and (2), which describe the LRS behavior based on ohmic conduction [41,42].

(1) $$J_{Ohmic}=\sigma E=q\mu N_{c}Eexp\left[\frac{-(E_{C}-E_{F})}{kT}\right]$$

(2) $$J_{Ohmic}\;\alpha \;E.exp\left(∸\frac{A}{T}\right)$$

where σ and m denote the electrical conductivity and mobility of the memory devices, respectively. Additionally, N_C_, E_C_ and E_F_ are the effective density of the states, and the conduction band and Fermi level of the functional layers.

In contrast, the primary conduction mechanism in the HRS of the memory device is Schottky emission, as is evident in Figure 4c. This conclusion is further supported by fitting curves across temperatures ranging from 300 K to 450 K, maintaining consistency with the Schottky emission model [43], as seen in Figure 4d.

(3) $$J=A^{*}T^{2}exp\;\left[\frac{-q\left(\phi_{b}-\sqrt{qV}\;/4\pi \varepsilon_{r}\varepsilon_{o}d\right)}{k_{B}T}\right]$$

where $A^{*}$ stands for the Richardson constant, d represents the dielectric layer’s thickness, $\phi_{b}$ is the Schottky barrier height, q denotes the electron charge, $\varepsilon_{r}$ signifies the permittivity of free space, $\varepsilon_{o}$ represents the vacuum permittivity, $k_{B}\;$ is the Boltzmann constant, and T is room temperature. Collectively, these outcomes indicate that the HRS conforms to the Schottky emission mechanism, thereby confirming the robust thermal stability of the memory device.

By simplifying the logarithm of the Schottky emission current equation, we can derive the curve ln(I/T^2^) ∝ V^(1/2) (results not presented here). This curve illustrates the relationship between the intercept and the slope, where the dependency of the barrier height and the Schottky distance can be founded. Subsequently, the slope and the intercept can be expressed as follows:

(4) $$slope\propto \;\sqrt{\frac{1}{\in_{i}d}}$$

(5) $$\left|Intercept\right|\propto \phi_{B}$$

Based on Equation (5), the Schottky barrier height is directly proportional to the intercept. It can be observed that the intercept value decreases when the temperature increased from 300 K to 450 K. Therefore, it can be deduced that the Schottky barrier height decreases with increasing temperature, and we determined its value to be 0.32 eV.

The observed behavior in the Pt/TiN/SnO_x_/Pt device structure implicates a filamentary RS mechanism. This hypothesis is supported by the current transport conduction mechanism fitting outcomes and the bipolar switching characteristics displayed by the SnO_x_-based memory devices. It is proposed that this behavior arises due to the formation of an interfacial layer effect, highlighted by the confirmed presence of a TiON interfacial layer through HRTEM analyses. This interfacial layer is found to be accountable for the enhanced switching properties observed.

**Figure 5:**
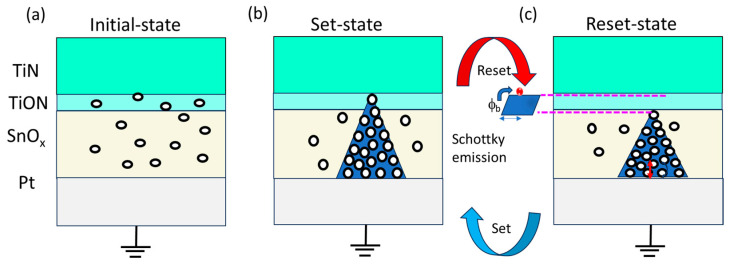
Possible bipolar switching mechanism of Pt/TiN/TiON/SnO_x_/Pt memory device. (**a**) Initial state, (**b**) set state, formation of conductive filaments, and (**c**) reset state, rupture of conductive filaments, respectively.

Based on the obtained results, a plausible switching mechanism is proposed, revolving around oxygen vacancies and oxygen ions, as depicted in Figure 5. This mechanism takes into account the work function disparities between the top TiN electrode (4.7 eV) and the bottom Pt electrode (5.65 eV), which influences the flow of electric charges and contributes to the manifestation of bipolar switching behaviors [44]. The electron affinity values for the TiON interface layer and the SnO_2_ switching layer are established to be 3.2 eV and 4.5 eV, respectively [45]. By calculating the work difference between metal electrodes and the electron affinity of oxide materials, the corresponding work function differences and electron affinity values are found to be 1.5 eV for the TiN/TiON interface and 1.15 eV for the Pt/SnO_x_ interface [46]. These calculations give rise to the formation of Schottky barrier heights at both interfaces.

The asymmetric behavior of the IV curve in the TiN/SnO_x_/Pt memory device points to the presence of a Schottky-like barrier at the TiN/TiON interface. TiN, known for its oxygen affinity, interacts with SnO2, leading to the creation of oxygen vacancies at the TiN/SnO_x_ interface as oxygen atoms are transferred from SnO_2_ to TiN [19]. This results in the random distribution of oxygen vacancies in the memory device’s pristine state. Consequently, a slightly higher voltage is required to initiate the formation of conductive filaments, as indicated in Figure 5a. Upon applying a positive voltage to the top TiN electrode, negatively charged oxygen ions migrate towards the TiN electrode, while oxygen vacancies are propelled by the electrical driving force. This culminates in the formation of conductive filaments within the switching layer, transitioning the memory device into a set state (Figure 5b). The concentration of oxygen vacancies in the SnO_2_ layer decreases from the BE to the TE, resulting in an oxygen vacancy gradient. This gradient fosters an additional driving force that propels oxygen vacancies from regions of high concentration to low concentration, reinforcing the transport of oxygen vacancies towards TiN [47]. During the reset process, oxygen ions either recombine with oxygen vacancies or move towards the Pt electrode. The additional oxygen vacancy driving force neutralizes a portion of the electrical driving force [48], leading to the rupture of conductive filaments and the reversion of the memory device to a high-resistance state (HRS), depicted in Figure 5c. Consequently, the reset voltage surpasses the set voltage, as illustrated in Figure 2d. Additionally, the TiON interfacial layer at the junction between TiN and SnO_x_ plays a significant role in containing resistive switching within its proximity. This mitigates the loss of oxygen ions and contributes to the device’s remarkable endurance and uniformity. Moreover, during the reset process, the TiON layer provides additional oxygen ions, which further lowers the voltage needed for the fracture of oxygen vacancy conductive filaments.

**Figure 6:**
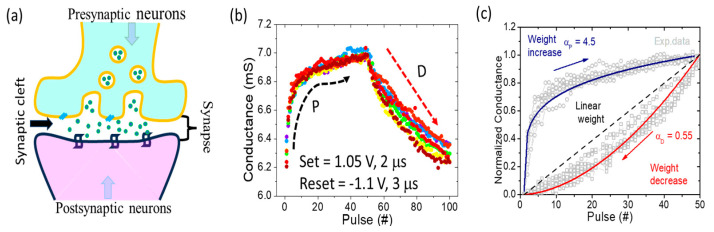
(**a**) Schematic demonstration of pre-synaptic and post-synaptic neuron-based synaptic functions. (**b**) Reproducibility of cycle-to-cycle (C2C) conductance modulation for potentiation and depression. (**c**) Evaluation of the nonlinearity in potentiation and depression synaptic plasticity with the fitting equations.

In the realm of practical applications involving electronic synaptic devices, their effective functioning relies on responding to pulsed input signals rather than the conventional cycles of direct current (DC) voltage sweeps. As a result, an exploration into the behavior of conductance variations in response to pulse cycling is imperative. Illustrated in Figure 6a is the conceptual representation of a biological neuron synapse [7,49,50]. When a pre-synaptic neuron is stimulated and its impulses are transmitted to a synaptic vesicle, these vesicles closely merge with the presynaptic membranes, resulting in their fusion and eventual rupture. This fusion releases neurotransmitters from the vesicles into the synaptic space, where they diffuse to reach the post-synaptic membrane, subsequently inducing either excitatory or inhibitory changes within the synaptic membrane.

In the context of the Pt/TiN/SnO_x_/Pt memory device, the stimulus applied to the presynaptic end corresponds to the source terminal of the memory device. The oxygen vacancies play a similar role to that played by neurotransmitters in biological synapses. By utilizing pulse voltages to generate stimulus signals, the result is an excitatory current resembling the presynaptic function. Consequently, manipulation of the memory device’s conductance becomes possible through the adjustment of pulse parameters such as width and amplitude, effectively tuning synaptic weights. Crucial parameters of memory devices, namely consecutive potentiation and depression, which determine the linearity and conductance range of synaptic weight update, can be adjusted accordingly. Figure 6b demonstrates the remarkable consistency of the memory device’s conductance modulation over ten consecutive cycles. Employing identical positive and negative pulse voltage waveforms, the synaptic behavior of the memory device is stimulated. Upon the application of 50 continuous positive (+1.05 V/2 μs) and negative (−1.1 V/3 μs) pulse voltages, a gradual shift towards long-term potentiation (LTP) and long-term depression (LTD) of conductance occurs. Figure 6c shows the nonlinearity factors of LTP and LTD characteristics which are extracted using the following equation [51]:

(6) G=GHRSα−GLRSα×w+GLRSα1α if α ≠0GLRSα×GHRSGLRSw if α≠0

where $G_{LRS}\;$ and $G_{HRS}\;$ represent the conductance of LRS and HRS, w is an internal variable (varied from 0 to 1), and $\alpha_{P}$,$\alpha_{D}\;$ are nonlinearity coefficients of potentiation and depression, respectively. The resulting nonlinearity values (α) for potentiation and depression of the memory devices are 4.5 and 0.55, indicating quasi-linear attributes. Notably, the linear trend of depression surpasses that of potentiation, consistent with abrupt resistance changes in the set state.

## 4. Conclusions

In conclusion, our study introduced a Pt/TiN/SnO_x_/Pt memory device as a promising avenue for advancing neuromorphic computing. Crafted through reactive sputtering, this device capitalizes on brain-inspired synaptic behavior facilitated by the TiON interface layer formed upon TiN-SnO_2_ interaction. Boasting an endurance of over 200 DC switching cycles, an ON/FFO ratio exceeding 20, and impressive multi-state retention, the SnO_x_-based device exhibits robust stability with minimal set and reset voltage variations of 9.89% and 3.2%, respectively. Controlled by negative reset voltage and compliance current, the device faithfully emulates synaptic characteristics. Structural analyses revealed a distinctive switching mechanism underpinned by an oxygen vacancy concentration gradient, complemented by current transport insights. Collectively, these findings underscore the transformative potential of SnO_x_-based memory devices, poised to revolutionize data storage and neuromorphic computing applications.

## Data Availability

Not applicable.
